# Association between gingival parameters and Oral health–related quality of life in Caribbean adults: a population-based cross-sectional study

**DOI:** 10.1186/s12903-019-0931-1

**Published:** 2019-11-01

**Authors:** J R Collins, A R Elías, M Brache, K Veras, G Ogando, M Toro, S Rivas-Tumanyan, A B Rajendra

**Affiliations:** 1 Department of Periodontology, School of Dentistry, Pontificia Universidad Católica Madre y Maestra (PUCMM-CSD), Santo Domingo, Dominican Republic; 2 School of Dental Medicine, Medical Sciences Campus, University of Puerto Rico, (UPR SDM), San Juan, Puerto Rico; 3 Department of Pathology, University of the West Indies Mona, Kingston, Jamaica

**Keywords:** Quality of life, Periodontal disease, OHIP-14 questionnaire, Caribbean, Adults

## Abstract

**Background:**

Good oral health has been associated with better quality of life and general health. In the Caribbean, there have been no studies regarding the association between oral health conditions and the quality of life of the population. The main purpose of this study was to investigate the association between gingival parameters and oral health–related quality of life (OHRQoL) in Caribbean adults. A secondary aim of the study was to gain more information on factors that impact OHRQoL in this population.

**Methods:**

This cross-sectional, epidemiological, population-based study was conducted in community settings. After the participants with missing Oral Health Impact Profile (OHIP) data were excluded, the sample size was 1821 (weighted according to the age and gender distribution in each target population). OHIP-14 standardized questionnaires were used to collect information. In addition, a medical/oral health questionnaire including sociodemographics, general health, dental visits, oral hygiene habits and knowledge, the frequency of dental visits, prosthesis use/hygiene, and smoking was administered. A multivariate model included predictors that showed significant associations in the univariate models. Odds ratios (ORs) and 95% confidence intervals (CIs) were reported; statistical significance was set at 0.05.

**Results:**

In the multivariate analysis, current smokers (OR = 2.34, 95% CI: 1.74–3.14 vs. never smokers), those who visited the dentist only when problems arose (OR = 1.65, 95% CI: 1.13–2.40 vs. those visiting once a year), and participants with any chronic disease/condition (OR = 1.38, 95% CI: 1.06–1.78) had higher odds of being in the highest tertile for OHIP score (poorer health).

**Conclusions:**

The present multicenter study identified potential modifiable risk factors for poor OHRQoL among adults in three Caribbean cities.

## Background

The World Health Organization (WHO) defines health as a state of complete physical, mental, and social well-being, and not only as the absence of disease or illness [1]. However, viewing health only from a perspective of deficiency, such as is the case when measures of morbidity and mortality are used, exclusively, ignores the fact that good health is much more than being alive and free of disease. Quality of life (QoL), a multidimensional concept, is determined with an individual’s subjective evaluation of both the positive and negative aspects of his or her life [2]. Oral health–related quality of life (OHRQoL) is a part of health-related QoL that focuses on oral health and orofacial concerns. Several diseases and oral conditions have been associated with the deterioration of QoL and, in general, with the systemic health of a given individual [3–6].

The Oral Health Impact Profile, comprising 14 items (OHIP-14), was developed to measure oral problems and their social impact, either as a total score index or in the seven following dimensions: functional limitation, physical pain, psychological discomfort, physical disability, psychological disability, social disability, and handicap [7, 8]. OHRQoL measures can be used when planning public health policies and assessing treatment needs for large populations, since modifying those factors has an influence on social and psychological well-being [9].

Studies in different regions have associated the deterioration of oral health with poor in adults, a critical issue since QoL worsens with age [10–12]. Dahl et al. [10], in a study conducted in Norwegian adults, found that self-rated oral health, frequency of dental visits, number of teeth, age, and gender were associated with having problems related to oral QoL in a multivariate analysis using the OHIP-14.

Gingival diseases (induced and not induced by dental biofilm) and periodontitis are inflammatory responses of the soft tissues surrounding the teeth and are a direct immune response to the establishment of a supra-subgingival biofilm. Periodontitis has recently been reported to likely have an impact on the QoL of patients [13, 14]. A study conducted on 443 Swedish adults showed that the effect of periodontal disease on QoL was considerable. In this study, individuals who experienced significant bone loss revealed that they had a reduced QoL compared to participants with less loss of supporting bone tissue [15].

In addition, relatively recent systematic reviews have suggested that periodontal disease may exert an impact on the QoL of individuals, with a greater severity of the disease being related to a greater impact. Additionally, the studies report that the recognition of the association was increased when full-mouth recording protocols were applied [16, 17].

Periodontitis has been directly associated with OHRQoL, and treatment of the disease may enhance QoL, from a patient’s perspective. However, PD remains prevalent, with little sign of improvement in terms of the severity of the disease [18–20].

PDs can cause symptoms such as bleeding gums, brushing pain, hypersensitivity, tooth mobility, and problems both with eating and with pronouncing words clearly, which can negatively affect OHRQoL, because such symptoms can compromise the daily life activities of the patient [19].

In Latin America and the Caribbean, the prevalence of PDs has been studied in adults and adolescents; these populations have high prevalences and severity of PDs [21–25]. Recently, an investigation was carried out in a group of 1847 adults to estimate the prevalence, severity, and associated risk factors of gingival inflammation in Kingston, Jamaica; Santo Domingo, Dominican Republic; and San Juan, Puerto Rico. The results showed that gingival inflammation was highly prevalent, with most of the participants presenting moderate or greater gingival inflammation (81.9%). Educational attainment, dental calculus, and a visible plaque index (VPI) > 30% were strongly associated with gingival inflammation [22].

Oral health has been a low priority for Caribbean local governments, and inadequate access to dental care provided by the public sector (with private-practice treatment being difficult to access for the most disadvantaged groups) has resulted in oral-health disparities in most of the countries in the region [26].

OHRQoL knowledge is limited worldwide. Values, goals, and socio-cultural expectations may differ among populations; therefore, reference values for the general population need to be defined.

Data on OHRQoL among Caribbean adults are scarce, and to our knowledge, there are no reports in adults that have gathered such data. The aim of the present study was to assess the association of gingival parameters with OHRQoL in Caribbean adults, as assessed by the OHIP-14. A secondary aim of the study was to gain more information on factors that impact OHRQoL.

## Methods

### Study design, study population and sampling

This research is part of a comprehensive cross-sectional study that assessed the prevalence, severity, and risk factors of gingival inflammation in Caribbean adults. In addition, the training and calibration of the examiners has been previously described [22].

This research was carried out in various communities of Kingston, Jamaica; Santo Domingo, Dominican Republic; and San Juan, Puerto Rico. Sampling procedures have been previously been published [22]. Briefly, the sample size of 611 individuals in each city was calculated assuming a gingivitis prevalence of 93.9% (average GI ≥ 0.5), 95% accuracy rate (confidence interval: 95%), a margin of error of 2%, with an oversample of 10%. Before the investigation began, the principal investigator and the clinical coordinator of the study in each of the 3 cities paid visits to the selected neighborhoods. These visits were conducted to select appropriate study settings and to distribute invitation flyers to residents.

In each city, 8 clusters (with 76–77 participants each) were selected using a systematic random sampling technique within neighborhoods of San Juan, Santo Domingo, and Kingston that were previously sorted according to their geographic distribution. Potential subjects from the general population who expressed an interest in participating and met the inclusion criteria (good general health, 18 years of age or older, presence of at least 4 permanent natural teeth), were consented into the study. Pregnant or breastfeeding women; subjects having undergone extensive prosthodontic treatment (partial removable dentures and/or fixed prosthodontics); wearers of orthodontic appliances (except retainers); and individuals presenting gingival purulent exudate, tooth mobility, and/or extensive loss of periodontal attachment or alveolar bone were excluded from the study. Participants needing prophylactic antibiotic therapy, on anticoagulant medication/treatment (except aspirin, but including nifedipine, cyclosporine, or phenytoin), or taking any other prescription medicines that might interfere with the study outcome were also excluded. Non-eligible candidates received a general oral screening and were offered oral health advice and referrals, as necessary.

### Data collection

After the participants signed the informed consent to participate in the study, two structured questionnaires (M/OH and OHIP) were administered to each participant by a trained interviewer. The M/OH collected information on socio-demographics, general health, dental visits, oral hygiene habits and knowledge, the frequency of dental visits, prosthesis use/hygiene, and smoking. The OHIP-14 questionnaire used in this study was previously developed by Slade (1997) [27] as a shorter version of the OHIP-49 questionnaire introduced by Locker and Miller (1994) [28]. The OHIP-14 questionnaire was used to measure OHRQoL; the Spanish version of the questionnaire has been validated previously [29, 30]. The overall OHIP score was calculated by adding points obtained from the answers to 14 QoL questions, which had the following options: never (0 points), rarely (1 point), sometimes (2 points), often (3 points), and always (4 points). The questionnaire was provided both in Spanish and in English to ensure comprehension by all the participants in the three countries.

After water-spraying, air-drying, and isolating the teeth with cotton rolls, a trained and calibrated evaluator made a six-point evaluation of each tooth in the entire mouth of each participant using a manual periodontal probe (UNC-15). The tooth surfaces were divided into three facial and three lingual regions, as follows: 1) mesio-facial, 2) mid-facial, 3) disto-facial, 4) mesio-lingual, 5) mid-lingual, and 6) disto-lingual. Periodontal clinical parameters were evaluated for all the teeth, excluding the third molars. VPI, Calculus Index (CI) and Gingival Index (GI) were evaluated on study participants. The visual plaque assessment was made based on the absence (0) or presence (1) of dental plaque. The Volpe–Manhold Index was used for calculus detection, and the Loe and Silness index (1963), as adapted from Talbott et al. [31], was used to evaluate gingival health. All the study parameters were entered in a password-protected computer during the examination. Participants who completed the interview and the clinical examination received advice about appropriate oral health care and were referred for oral/dental treatment, as required.

### Ethical considerations

The Good Clinical Practice standards were applied and maintained throughout the study.

Locations such as hospitals and commercial establishments were not included in the study. The protocol used for this study is in accordance with the Declaration of Helsinki and was reviewed and approved by the following Institutional Review Boards and National Bioethics Boards of each country: the Advisory Panel on Ethics and Legal-Medical Affairs, University of West Indies; the Ministry of Health of Jamaica, Kingston, Jamaica (Protocol #248, 15/16); the Consejo Nacional de Bioética en Salud (CONABIOS) Dominican Republic, Santo Domingo (Protocol #042–2016); and the Institutional Review Board of the University of Puerto Rico, San Juan (Protocol #360216).

### Statistical analysis

All the observations were weighted according to the distribution of age and gender in each target population. The weights were later normalized and adjusted to represent an equal number (610) of participants in each location. After the exclusion of the participants who did not complete the OHIP questionnaire, the weighted sample size for the descriptive analysis of QoL was set at 1821. Descriptive statistics were employed to estimate the summary OHIP score, which was analyzed in two different formats: (a) as a summary score, and (b) grouped in tertiles, with the upper third tertile corresponding to the worst levels of QoL. The distribution of the summary OHIP score and its tertiles was compared across the categories of potential predictors. Univariate associations between potential predictors and the tertiles of OHIP scores were evaluated using multinomial logistic regression, using the lowest tertile as the reference. Ordinal logistic regression analysis was also considered; however, due to violation of proportional odds assumption for some predictors, multinomial regression was preferred. The associations between the same predictors and the summary OHIP score were evaluated using negative binomial regression models, due to the distribution of the summary OHIP score variable. Participants with missing values for smoking (*N* = 3), level of education (*N* = 4), frequency of tooth brushing (*N* = 6), and calculus (*N* = 2) were excluded from the regression analysis, with a final weighted sample size for multivariate analysis of 1807 participants; the participants with missing information on frequency of dental visits were grouped under a separate category (*N* = 81). In addition to age, the predictors that demonstrated statistically significant associations in the univariate models were included in the multivariate analysis. To select the clinical variables (gingivitis, plaque, calculus) to be included in the final multivariate model, we explored different combinations of those variables, and the model with the lowest Schwarz and Akaike information criteria and the highest R-square was selected. The final multivariate model included age (in years), gender, smoking status, education, prior diagnosis of any major disease/condition, number of missing teeth, mean gingivitis score, plaque and calculus indices, frequency of dental visits, use of dental floss, and city of residence (location). We additionally tested for interactions between demographic variables (age, gender) and oral health indicators in our multivariate models, using Wald-type *p*-values for the interaction terms. Negative binomial regression coefficients, standard errors, and the corresponding exponentiated coefficients (95% confidence intervals) were reported for count data (summary OHIP scores); odds ratios (ORs and 95% confidence intervals) were reported for the OHIP tertile analysis.

All descriptive and multinomial logistic regression analyses were conducted using SAS software, Version 9.3 (SAS Institute, Cary, NC), with a 0.05 statistical significance level, using two-sided tests, and accounted for survey weights and clustering effects. Negative binomial regression analysis was conducted using Stata Statistical Software, Release 13.1 (College Station, TX: StataCorp LP), which allows this kind of analysis, while accounting for clustering effects and adjusting for survey weights in these models.

## Results

A total of 1839 adults (weighted *N* = 1821) aged 18 to 96 years, with a mean of 37.0 ± 9.03 years, from Santo Domingo, San Juan, and Kingston were included in this study. Males represented 45.9% and females 54.1% of the overall study participants, and most of the participants were in the 50 years old or older age group. The summary OHIP score ranged from 0 to 53, with a mean of 7.2 (standard error: 0.2) and a median of 4 (interquartile range: 1.13–9.54). Sociodemographic and relevant characteristics are presented in Table 1, which shows that the majority of the participants had a middle/technical level of education (54.69%), were never smokers (64.47%), did not have any systemic diseases (62.33%), went to the dentist in emergencies only (59.31%), and reported regular (twice per day) tooth brushing (62.77%); less than half used dental floss regularly (40.36%). With respect to periodontal parameters, Table 2 also shows that more than half of the participants had a high prevalence of gingival bleeding at ≥40% of the probing sites (52.55%), and almost all presented a visible plaque index ≥30% (96.98%).

**Table 1:** Distribution of the summary OHIP score among all participants, and by location

OHIP score Percentile	Among all (WtN = 1821)	Kingston, Jamaica (WtN = 603)	Santo Domingo, DR (WtN = 608)	San Juan, PR (WtN = 610)
P0 (min)	0.00	0.00	0.00	0.00
P10	0.00	0.00	0.00	0.00
P20	0.12	1.02	0.00	0.00
P30	1.53	1.73	1.58	1.22
P40	3.01	3.24	3.24	2.26
P50	4.00	4.83	4.51	3.58
P60	5.80	6.14	5.97	5.21
P70	8.00	8.78	9.18	7.10
P80	11.53	11.69	13.11	9.49
P90	17.33	17.20	18.95	14.78
P100 (max)	53.00	53.00	48.00	45.00

**Table 2:** Mean (Standard Error around the mean), and median (Interquartile range) for the summary OHIP score according to categories of exposures; distribution of exposures according to the tertiles of quality of life score, among all participants (weighted *N* = 1821)

	WtN	Mean (SE)	Median (Q1-Q3)	Tertile 1 WtN(%)	Tertile 2 WtN(%)	Tertile 3 WtN(%)
Among all	1821	7.20 (0.20)	4.00 (1.13–9.54)	652 (100%)	533 (100%)	636 (100%)
Age group						
18–19 years	93	6.58 (0.64)	3.95 (1.45–9.34)	28 (4.25%)	34 (6.45%)	31 (4.86%)
20–29 years	449	6.83 (0.36)	3.63 (0.87–8.95)	175 (26.81%)	129 (24.15%)	146 (22.92%)
30–39 years	429	6.94 (0.49)	3.97 (1.13–9.03)	160 (24.55%)	126 (23.62%)	143 (22.47%)
40–49 years	337	7.63 (0.51)	5.04 (1.37–10.02)	110 (16.81%)	103 (19.26%)	125 (19.62%)
50 years or older	513	7.55 (0.26)	4.30 (1.09–10.05)	180 (27.58%)	141 (26.52%)	192 (30.13%)
Gender						
Male	837	6.29 (0.29)	3.51 (0.82–8.04)	333 (51.08%)	249 (46.79%)	254 (40.00%)
Female	984	7.96 (0.28)	5.14 (1.32–11.19)	319 (48.92%)	284 (53.21%)	381 (60.00%)
Level of education						
None/basic	265	8.89 (0.47)	0.43 (1.48–12.56)	79 (12.11%)	70 (13.09%)	116 (18.30%)
Middle/technical	996	7.25 (0.22)	3.98 (1.20–9.73)	348 (53.38%)	296 (55.56%)	351 (55.28%)
University	557	6.31 (0.32)	3.61 (0.81–7.89)	229 (34.19%)	167 (31.34%)	167 (26.27%)
Missing	3	2.57 (2.62)	–	2 (0.31%)	0	1 (0.16%)
Smoking						
Current	361	8.79 (0.44)	5.63 (1.78–11.85)	99 (15.19%)	100 (18.70%)	163 (25.59%)
Past	283	6.71 (0.54)	3.79 (0.86–9.19)	108 (16.51%)	83 (15.58%)	92 (14.47%)
Never	1174	6.84 (0.24)	3.83 (1.01–8.90)	442 (67.84%)	350 (65.73%)	381 (59.94%)
Missing	3	1.38 (0.64)	–	3 (0.46%)	0	0
Any diseases/conditions						
Yes	686	8.03 (0.35)	5.01 (1.24–11.14)	231 (35.42%)	184 (34.52%)	271 (42.62%)
No	1135	6.69 (0.28)	3.79 (1.07–8.81)			
Diabetes diagnosis						
Yes	137	9.17 (0.72)	5.30 (1.58–11.46)	43 (6.58%)	37 (6.92%)	57 (9.01%)
No	1683	7.03 (0.21)	3.93 (1.08–9.43)			
Hypertension diagnosis						
Yes	365	7.61 (0.38)	4.81 (1.16–10.23)	122 (18.73%)	102 (19.22%)	141 (22.12%)
No	1455	7.09 (0.23)	3.91 (1.12–9.33)			
Frequency of dental visits						
≥ Once per year	600	6.03 (0.46)	3.32 (0.39–7.78)	240 (36.88%)	191 (35.79%)	169 (26.56%)
Never	60	7.45 (0.98)	2.85 (0–11.46)	27 (4.21%)	12 (2.27%)	21 (3.24%)
Only when there is a problem	1080	7.82 (0.22)	5.15 (1.39–11.01)	351 (53.80%)	312 (58.55%)	416 (65.48%)
Missing	81	7.30 (0.93)	3.55 (0.68–9.62)	33 (5.11%)	18 (3.38%)	30 (4.72%)
Frequency of tooth brushing						
≥ Three times per day	472	7.52 (0.33)	4.11 (1.09–9.84)	170 (26.09%)	129 (24.27%)	172 (27.12%)
Twice per day	1143	6.85 (0.28)	3.91 (1.14–9.07)	413 (63.42%)	357 (67.00%)	372 (58.54%)
Once per day	188	8.13 (0.67)	5.22 (1.09–11.69)	64 (9.85%)	40 (7.46%)	85 (13.29%)
< Once per day	12	9.19 (2.41)	6.02 (3.01–10.18)	2 (0.29%)	6 (1.09%)	4 (0.60%)
Missing	6	14.54 (7.55)	4.03 (0–22.55)	2 (0.31%)	1 (0.19%)	3 (0.47%)
Use of dental floss						
Yes	735	6.30 (0.29)	3.64 (1.02–8.19)	282 (43.26%)	227 (42.63%)	226 (35.52%)
No	1086	7.81 (0.22)	4.91 (1.22–10.70)			
Location						
Kingston	603	7.42 (0.18)	4.83 (1.37–9.86)	204 (31.33%)	175 (32.81%)	224 (35.17%)
Santo Domingo	608	7.75 (0.27)	4.51 (1.17–11.25)	214 (32.78%)	168 (31.51%)	226 (35.58%)
San Juan	610	6.43 (0.41)	3.58 (0.60–8.16)	234 (35.88%)	190 (35.68%)	186 (29.25%)
Mean gingival index, mean ± SE		–	–	1.41 ± 0.04	1.47 ± 0.05	1.58 ± 0.05
Mean interproximal gingival index, mean ± SE		–	–	1.44 ± 0.04	1.50 ± 0.05	1.60 ± 0.05
Gingival bleeding at ≥ 40% of the probing sites						
Yes	957	8.11 (0.26)	5.16 (1.34–11.30)	307 (47.12%)	269 (50.53%)	380 (59.79%)
No	864	6.19 (0.30)	3.54 (0.84–7.94)			
Mean plaque index, mean ± SE		–	–	0.92 ± 0.02	0.93 ± 0.01	0.95 ± 0.01
Mean interproximal plaque index, mean ± SE		–	–	0.95 ± 0.02	0.96 ± 0.01	0.97 ± 0.01
Visible plaque index (plaque index ≥ 30%)						
Yes	1766	7.23 (0.21)	4.10 (1.14–9.60)	625 (95.90%)	519 (97.36%)	622 (97.90%)
No	54	6.02 (1.06)	2.36 (0.90–6.93)			
Mean calculus index, mean ± SE		–	–	0.63 ± 0.03	0.66 ± 0.04	0.69 ± 0.04
Mean interproximal calculus index, mean ± SE		–	–	0.66 ± 0.03	0.70 ± 0.04	0.72 ± 0.03

### OHIP score tertile analysis

In the unadjusted analysis (Table 3), being male and use of dental floss was associated with lower odds of reporting a low OHRQoL (tertile 3 vs. 1). Lower levels of education, current smoking, major disease diagnoses, and visiting the dentist only when there was a problem, on the other hand, were associated with higher odds of reporting a low OHRQoL. Compared to participants from San Juan, those from Kingston and Santo Domingo were more likely to be in the worst tertile for OHRQoL in the univariate analysis. All the clinical variables for oral health (gingival, plaque, and calculus variables, and number of missing teeth) were significantly associated with low OHRQoL. In the univariate analysis, participant age, tooth brushing frequency, and a diagnosis of diabetes or hypertension were not associated with self-reported OHRQoL.

**Table 3:** Univariate odds ratios (ORs) and 95% confidence intervals (CIs) for tertiles of quality of life score (lowest tertile was used as the reference), according to categories of potential predictors^a^, among all participants (Weighted *N* = 1821)

Predictors	Tertile 2 vs. Tertile 1 (ref.)		Tertile 3 vs. Tertile 1 (ref.)	
	OR (95% CI)	*p*-value	OR (95% CI)	*p*-value
Age, years	1.00 (0.99; 1.01)	0.725	1.00 (0.99; 1.01)	0.411
Gender				
Male	0.84 (0.70; 1.01)	0.063	0.64 (0.50; 0.82)	< 0.001
Female (ref.)	1.0	–	1.0	–
Level of education (WtN = 1817)^b^				
None/basic	1.18 (0.80; 1.73)	0.401	1.97 (1.38, 2.79)	< 0.001
Middle/technical	1.14 (0.84; 1.54)	0.419	1.35 (1.06; 1.72)	0.017
University (ref.)	1.0	–	1.0	–
Smoking (WtN = 1818)^b^				
Current	1.27 (0.95; 1.69)	0.102	1.91 (1.45; 2.50)	< 0.001
Past	0.97 (0.73; 1.31)	0.859	0.99 (0.72; 1.37)	0.960
Never (ref.)	1.0	–	1.0	–
Any diseases/conditions				
Yes	0.96 (0.75; 1.23)	0.752	1.35 (1.10; 1.66)	0.004
No (ref.)	1.0	–	1.0	–
Diabetes				
Yes	1.06 (0.73; 1.53)	0.772	1.41 (1.00; 1.99)	0.053
No (ref.)	1.0	–	1.0	–
Hypertension				
Yes	1.03 (0.75; 1.42)	0.845	1.23 (0.96; 1.58)	0.102
No (ref.)	1.0	–	1.0	–
Number of missing teeth	1.03 (0.99;1.06)	0.055	1.07 (1.04; 1.09)	< 0.001
Mean gingival index	1.45 (1.01; 2.07)	0.044	2.41 (1.91; 3.05)	< 0.001
Mean interproximal gingival index	1.45 (1.02; 2.07)	0.041	2.40 (1.89; 3.04)	< 0.001
Gingival bleeding at ≥ 40% of probing sites				
Yes	1.15 (0.86; 1.53)	0.353	1.67 (1.31; 2.13)	< 0.001
No (ref.)	1.0	–	1.0	–
Mean plaque index	1.41 (0.76; 2.61)	0.279	3.33 (1.65; 6.70)	< 0.001
Mean interproximal plaque index	1.50 (0.73; 3.11)	0.272	2.27 (1.26; 4.07)	0.006
Visible plaque index (plaque index ≥ 30%)				
Yes	1.57 (0.77; 3.22)	0.214	2.00 (1.16; 3.43)	0.013
No (ref.)	1.0	–	1.0	–
Mean calculus index (WtN = 1819)^b^	1.27 (0.91; 1.78)	0.159	1.58 (1.16; 2.15)	0.004
Mean interproximal calculus index (WtN = 1819)^b^	1.32 (0.91; 1.92)	0.141	1.55 (1.14; 2.11)	0.005
Frequency of dental visits				
≥ Once per year (ref.)	1.0	–	1.0	–
Never	0.56 (0.31; 1.00)	0.049	1.07 (0.55; 2.10)	0.847
Only when there is a problem	1.12 (0.79; 1.58)	0.515	1.59 (1.25; 2.29)	< 0.001
Missing	0.68 (0.36; 1.29)	0.239	0.28 (0.83; 1.98)	0.264
Frequency of tooth brushing (WtN = 1815)^b^				
≥ Three times per day	0.25 (0.05; 1.31)	0.100	0.50 (0.09; 2.83)	0.432
Twice per day	0.28 (0.06; 1.38)	0.118	0.44 (0.08; 2.55)	0.362
Once per day	0.20 (0.04; 1.06)	0.058	0.65 (0.13; 3.35)	0.605
< Once per day (ref.)	1.0	–	1.0	–
Use of dental floss				
Yes	0.98 (0.77; 1.23)	0.831	0.72 (0.59; 0.89)	0.002
No (ref.)	1.0	–	1.0	–
Location				
Kingston	1.05 (0.78; 1.42)	0.737	1.38 (1.04; 1.83)	0.026
Santo Domingo	0.97 (0.70 l 1.34)	0.838	1.33 (1.03; 1.72)	0.029
San Juan (ref.)	1.0	–	1.0	–

^a^Odds ratio estimates were obtained from multinomial logistic regression models, with each predictor separately and the three-level categorical OHIP score (tertile) as the outcome, using the lowest tertile (best summary quality of life score) as the reference

^b^For these models, only participants with non-missing information on the predictor were included

After adjusting for variables in the multivariate model (Table 4), the association between participant gender and OHRQoL remained statistically significant, with males having half the odds of being in the worst OHRQoL tertile compared to females (tertile 3 vs. 1, OR_male vs. female_ = 0.54, 95% CI: 0.42–0.68). Current smokers had more than twice the odds of being in the highest tertile (i.e., worst OHRQoL) compared to never smokers (OR_current vs. never_ = 2.13, 95%: 1.57–2.88). The associations between having a prior diagnosis of any major disease/condition, visiting the dentist only when there is a problem, and OHRQoL (as the outcome) maintained their statistical significance and magnitude in the multivariate analysis (OR_any disease diagnosis_ = 1.39, 95% CI: 1.10–1.76; OR_only visiting dentist when there is problem vs. visiting ≥ 1 year_ = 1.58, 95% CI: 1.07–2.32). The OR estimates for the level of education and use of dental floss, on the other hand, attenuated and were no longer significant after adjusting for other variables in the model. Residents of Kingston had significantly lower odds of reporting worse OHRQOL (tertiles 2 and 3), compared to those from San Juan. Out of the clinical oral health indicators, the number of missing teeth andmean G-Index remained significantly associated with being in the worst tertile of OHRQoL. In interaction analysis, only the interaction between age (in years) and number of missing teeth was statistically significant for comparison between the highest and lowest OHIP tertiles, with the OR of 0.998 (95% CI: 0.996–0.999); inclusion of the interaction term did not significantly change the estimates for variables other than age and number of missing teeth (Additional file 1: Table S1).

**Table 4:** Multivariate odds ratios (ORs) and 95% confidence intervals (CIs) for tertiles of quality of life score (lowest tertile was used as the reference), according to predictors^a^, among all participants (Weighted *N* = 1807)

Predictors	Tertile 2 vs. Tertile 1 (ref.)		Tertile 3 vs. Tertile 1 (ref.)	
	OR (95% CI)	*p*-value	OR (95% CI)	*p*-value
Age, years	0.99 (0.98; 0.99)	0.027	0.99 (0.98; 0.99)	0.002
Gender				
Male	0.79 (0.64; 0.98)	0.032	0.54 (0.42; 0.68)	< 0.001
Female (ref.)	1.0	–	1.0	–
Level of education				
None/basic	1.08 (0.76; 1.53)	0.666	1.14 (0.76; 1.71)	0.519
Middle/technical	1.11 (0.81; 1.51)	0.517	1.04 (0.76; 1.42)	0.820
University (ref.)	1.0	–	1.0	–
Smoking				
Current	1.24 (0.85; 1.83)	0.264	2.13 (1.57; 2.88)	< 0.001
Past	1.01 (0.75; 1.35)	0.950	1.02 (0.69; 1.49)	0.930
Never (ref.)	1.0	–	1.0	–
Any diseases/conditions				
Yes	0.93 (0.74; 1.16)	0.512	1.39 (1.10; 1.76)	0.005
No (ref.)	1.0	–	1.0	–
Number of missing teeth	1.04 (1.01; 1.07)	0.019	1.05 (1.02; 1.08)	< 0.001
Mean gingival index	1.52 (1.10; 2.11)	0.012	2.24 (1.69; 2.97)	< 0.001
Mean plaque index	1.00 (0.53; 1.89)	0.992	1.63 (0.81; 3.29)	0.175
Mean calculus index	1.13 (0.73; 1.73)	0.591	0.92 (0.62; 1.35)	0.663
Frequency of dental visits				
≥ Once per year (ref.)	1.0	–	1.0	–
Never	0.54 (0.27; 1.06)	0.073	1.02 (0.46; 2.25)	0.963
Only when there is a problem	1.09 (0.71; 1.67)	0.687	1.58 (1.07; 2.32)	0.020
Missing	0.68 (0.33; 1.41)	0.300	1.39 (0.86; 2.24)	0.184
Use of dental floss				
Yes	1.04 (0.81; 1.35)	0.745	0.96 (0.70; 1.31)	0.790
No (ref.)	1.0	–	1.0	–
Location				
Kingston	0.70 (0.51; 0.95)	0.020	0.57 (0.34; 0.94)	0.028
Santo Domingo	0.76 (0.54; 1.08)	0.127	0.83 (0.57; 1.19)	0.311
San Juan (ref.)	1.0	–	1.0	–

^a^Odds ratio estimates were obtained from a multinomial logistic regression model, with all listed variables as predictors and the three-level categorical OHIP score (tertile) as the outcome, using the lowest tertile (best summary quality of life score) as the reference

### Analysis of OHIP summary score summary OHIP score analysis

In the univariate analysis (Table 5), several factors were associated with OHIP score: males appeared to have lower OHIP scores compared to females, whereas lower levels of education were associated with higher scores (both none/basic and midlevel/technical education vs. university). Similarly, those who were smokers (current vs. never) and who had any major diseases/conditions as well as a diabetes diagnosis, or those visiting their dentist only when they had a problem (vs. visiting ≥ once a year) had higher OHIP scores. Residents of Kingston and Santo Domingo had, on average, higher OHIP scores compared to the participants from San Juan. When it came to clinical indicators of oral health, the number of missing teeth, all gingival and calculus indices, and mean plaque index were positively associated with the OHIP scores (*p* < 0.05), with higher oral indices (worse oral health) being associated with higher OHIP scores. In the multivariate analysis (Table 6), several of the considered predictors (such as gender, current smoking, presence of any major condition/diagnosis, number of missing teeth, and G-Index) retained their association with the OHIP scores, whereas others (education, plaque and calculus indices, use of dental floss) attenuated and lost statistical significance. There were no statistically significant interactions identified in the analysis of the summary OHIP scores.

**Table 5:** Univariate negative binomial regression coefficients (b) and their standard errors, for the summary quality of life score, according to categories of potential predictors*, among all participants (Weighted *N* = 1821)

Predictors	b	SE	*p*-value
Age, years	0.002	0.002	0.250
Gender			
Male vs. Female (ref.)	−0.236	0.052	< 0.001
Level of education (WtN = 1817)			
None/basic	0.343	0.080	< 0.001
Middle/technical	0.140	0.061	0.022
University (ref.)	–	–	–
Smoking (WtN = 1818)			
Current	0.251	0.062	< 0.001
Past	−0.019	0.074	0.795
Never (ref.)	–	–	–
Any diseases/conditions			
Yes vs. No (ref.)	0.182	0.053	0.001
Diabetes			
Yes vs. No (ref.)	0.266	0.010	0.007
Hypertension			
Yes vs. No (ref.)	0.071	0.064	0.262
Number of missing teeth	0.036	0.005	< 0.001
Mean gingival index	0.418	0.056	< 0.001
Mean interproximal gingival index	0.407	0.056	< 0.001
Gingival bleeding at ≥ 40% of probing sites			
Yes vs. No (ref.)	0.270	0.052	< 0.001
Mean plaque index	0.436	0.188	0.021
Mean interproximal plaque index	0.240	0.188	0.203
Visible plaque index (plaque index ≥ 30%)			
Yes vs. No (ref.)	0.184	0.186	0.323
Mean calculus index (WtN = 1819)	0.206	0.072	0.005
Mean interproximal calculus index (WtN = 1819)	0.202	0.074	0.007
Frequency of dental visits			
≥ Once per year (ref.)	–	–	–
Never	0.212	0.164	0.197
Only when there is a problem	0.261	0.058	< 0.001
Missing	0.190	0.139	0.172
Frequency of tooth brushing (WtN = 1815)			
≥ Three times per day	−0.200	0.263	0.446
Twice per day	− 0.294	0.260	0.258
Once per day	− 0.126	0.268	0.648
< Once per day (ref.)	–	–	–
Use of dental floss			
Yes vs. No (ref.)	−0.214	0.053	< 0.001
Location			
Kingston	0.143	0.064	0.026
Santo Domingo	0.186	0.065	0.004
San Juan (ref.)	–	–	–

**Table 6:** Multivariate negative binomial regression coefficients (b), their standard errors, exponentiated coefficients with 95% confidence intervals (CI) for the summary quality of life score, according to categories of potential predictors*, among all participants (Weighted *N* = 1807)

Predictors	b	SE	*p*-value	exp(b)	95% CI
Age, years	−0.008	0.002	0.001	0.992	(0.988; 0.997)
Gender					
Male vs. Female (ref.)	−0.263	0.053	< 0.001	0.769	(0.693; 0.853)
Level of education					
None/basic	0.019	0.094	0.840	1.019	(0.847; 1.227)
Middle/technical	−0.017	0.068	0.799	0.983	(0.860; 1.123)
University (ref.)	–	–	–		
Smoking					
Current	0.287	0.068	< 0.001	1.332	(1.166; 1.522)
Past	−0.002	0.076	0.974	0.998	(0.860; 1.157)
Never (ref.)	–	–	–	–	–
Any diseases/conditions					
Yes vs. No (ref.)	0.187	0.057	0.001	1.205	(1.078; 1.348)
Number of missing teeth	0.032	0.006	< 0.001	1.033	(1.020; 1.045)
Mean gingival index	0.367	0.070	< 0.001	1.444	(1.259; 1.656)
Mean plaque index	−0.012	0.197	0.951	0.988	(0.672; 1.453)
Mean calculus index	−0.008	0.089	0.925	0.992	(0.833; 1.180)
Frequency of dental visits					
≥ Once per year (ref.)	–	–	–	–	–
Never	0.131	0.160	0.411	1.140	(0.833; 1.180)
Only when there is a problem	0.211	0.073	0.004	1.234	(1.071; 1.423)
Missing	0.201	0.140	0.151	1.223	(0.929; 1.610)
Use of dental floss					
Yes vs. No (ref.)	−0.061	0.059	0.301	0.941	(0.838; 1.056)
Location					
Kingston	−0.304	0.094	0.001	0.738	(0.613; 0.888)
Santo Domingo	−0.039	0.080	0.625	0.962	(0.822; 1.125)
San Juan (ref.)	–	–	–	–	–

## Discussion

The present cross-sectional multicenter study comprised 1821 adults randomly selected from Kingston (Jamaica), Santo Domingo (Dominican Republic), and San Juan (Puerto Rico), with a mean age of 37.0 ± 9.03 years. To date, there are no published data regarding the association between periodontal status and OHRQoL in a representative sample of adult populations from these three cities in the Caribbean Basin.

Investigators using data gathered by the OHIP-14 questionnaire have been able to significantly predict periodontal symptoms, including swollen, sore, and receding gums, as well as toothache, loose teeth, and bad breath [6, 32]. This study mentioned above showed that periodontal conditions had a significant impact on OHRQoL.

In the present study, upon comparing the frequency distribution of gingival bleeding at ≥40% of the probing sites (according to the tertiles of the QoL score) among all the participants, the groups with higher prevalence of bleeding sites were in the highest tertile, presenting a greater impact on QoL. Additionally, having high gingival, plaque, and calculus indices was statistically significantly associated with being in the highest tertile (worst QoL). However, when the periodontal variables were analyzed in the multivariate statistical model, only an elevated G-Index and not the presence of plaque or calculus continued to show a statistical significance associated with high tertiles.

These results are consistent with those of other studies reporting that periodontal disease and conditions are associated with a negative impact on QoL [33–36]. In a recent systematic review, Ferreira et al. [16] reported that gingivitis was associated with pain, difficulties performing oral hygiene and wearing dentures. Additionally, they found that gingivitis was also negatively correlated with comfort. The results of our study indicate that periodontal disease may exert an impact on the QoL of individuals, with a greater severity of disease related to a greater impact. This suggests that the provision of periodontal treatment to the Caribbean adult population can greatly improve their QoL. Shanbhag et al. [37] demonstrated that nonsurgical periodontal therapy had a greater impact on OHRQoL than surgical therapy did and that poor clinical response to therapy was correlated to poor OHRQoL outcomes. In addition, Goel et al. [20] concluded that PD is directly associated with OHRQoL and that treatment of the disease may enhance QoL from a patient’s perspective. They reported that scaling and subgingival root planing had a better influence on OHRQoL than did supragingival scaling. Other studies have shown that a patient’s perception of OHRQoL improved with periodontal nonsurgical therapy, particularly after the supragingival treatment, suggesting that this intervention might be considered important to the reduction of the negative impact on OHRQoL [38, 39].

The results of the multivariate regression analyses of our study showed no significant relationship between age, and OHRQoL, being equal in young and old adult populations. However, it has been shown that as age progresses, certain conditions, such as loss of income, the presence of a chronic disease, and exclusion from health programs, have been reported to influence health and QoL [40, 41]. Åstrøm et al. analysed longterm effect of socio-behavioral characteristics on oral impact and concluded that disadvantaged socio-behavioral conditions at age fifty had a long lasting detrimental effect on QHRQoL at age sixty-five [42]. Therefore, the authors recommended early protection against the effect of socio-behavioral adversity by imposing economic barriers, ensuring the provision of high-quality care and the promotion of healthy lifestyles, both of which appear to have the potential to reduce the negative impact of poor oral health on older individuals.

In a recent study in India, Sanadhya et al. [43] showed that OHIP-14 scores were significantly associated with age, gender, and place of residency. In our study, when predictors were evaluated separately in the univariate model, women and people living in Santo Domingo had a significantly higher probability of being in the third tertile for QoL than did people of either sex and those residing in San Juan or Kingston. However, when the multinomial logistic regression model was analyzed, gender maintained statistical significance, but the association between place of residency and OHRQoL was not statistically significant. Taking into account that several studies have shown that individuals living in rural areas have poorer OHRQoL scores in three main categories (physical pain, psychological discomfort, and social disability), we recommend that longitudinal studies that can analyze the populations that live in rural and urban areas be implemented. In a study by Gaber et al. [44], the authors estimated the rural–urban disparity in the OHRQoL of the Quebec adult population. They found potential differences in OHRQoL of Quebecoise rural and urban populations. Additionally, the results showed that rural residents were twice as far from dental care services than were their urban counterparts and that they were also less motivated to seek regular dental check-ups. In another study, Espinoza et al. [45] conducted a study in 3050 Chilean adults to investigate the socioeconomic disparities in oral health. The participants showed a significant association of having relatively poorer OHRQoL with being female, living in a rural area, and self-reporting a generally poor QoL; these parameters were subsequently controlled for in the multivariate analysis.

Health behavior is defined as activities promoting, protecting, or maintaining the health of the individual, while risk behavior relates to actions with negative effects on human health [46]. The frequency of visits to the dentist has been related to QoL, in recent publications [47]. The present study demonstrates that individuals who never visit unless or only visit the dentist when there is a problem are associated with worse QoL than are their dentist-visiting counterparts. Furthermore, our findings showed that for health-related behaviors associated with lifestyle (such as smoking), less frequent smoking was significantly associated with a better QoL. These findings were similar to those from a cross-sectional study that evaluated the impact of health-related behaviors and dental attendance on OHRQoL in individuals attending two central dental clinics in Israel for conservative dental treatment [47]. The investigators found that the practicing of certain health behaviors, all of which conferred significant health benefits, and which behaviors include not consuming alcohol, regular physical activity, smoking fewer packs of cigarettes per year, and making routine dental visits, have a protective effect on OHRQoL. It has been shown that smoking influences the general health of the individual, and smoking has also been associated with psychosocial disorders that consequently have a negative effect on QoL [48]. In our study, when we compared individuals who reported that they used dental floss frequently with those who did not, we found that the former were more often in the lowest tertile in the univariate model than were the latter (*p* ≤ 0.01). It is possible that individuals who use dental floss value their oral health more highly and consequently experience a better QoL; however, this association cannot be found in the multivariate analysis.

In spite of the fact that this is the first report on the association between the periodontal status of Caribbean adults and their OHRQoL in a representative sample of adults from three cities of the Caribbean Basin, there are some limitations of our study. The study was carried out in urban areas; therefore, the results cannot be generalized to the rural areas near the studied cities or to those of other Caribbean countries. Due to time constraints, caries/restorations (local risk factors for gingival inflammation) [49], were not recorded.

Further nationwide studies, including those meant to gather and analyze caries/restoration data, should be conducted in other Caribbean countries. Moreover, the health-related behavior data were self-reported and are therefore not verifiable. Other investigations will have to be carried out to determine oral health, taking into account the unique characteristics of each country and the relationships of those characteristics to the QoL of individuals residing in rural areas.

## Conclusions

The present multi-city study, the first to assess the impact of OHRQoL on a Caribbean population, identified potential modifiable risk factors for a poor OHRQoL in adults from three Caribbean cities. These predictors deteriorate and significantly affect QoL, which highlights the need for the development by public health officials, oral healthcare professionals, educators, and relatives of strategies to effectively promote good oral health and eliminate or control detrimental oral-health behaviors, specifically for Caribbean residents. Further studies are needed to confirm these results.

## Supplementary information

**Additional file 1: Table S1.** Multivariate odds ratios (ORs) and 95% confidence intervals (CIs) for tertiles of quality of life score (lowest tertile was used as the reference), according to predictors* and the interaction between age and number of teeth, among all participants (Weighted *N* = 1807). Results of regression analysis on tertiles of quality of life score.

## Data Availability

The dataset will be made available to reader(s) from corresponding author when a justified request from a reader is received. The data of the three countries that participated in this multicentric study are stored at the Faculty of Health Sciences of the University of Puerto Rico, Medical Sciences Campus. Although each country maintains its data and written documentation, individually.

## Abbreviations

OHIP: Oral Health Impact Profile
OHRQoL: Oral health–related quality of life
QoL: Quality of life
VPI: Visible plaque index
