# Supplementary material for: Association between gingival parameters and Oral health–related quality of life in Caribbean adults: a population-based cross-sectional study
Source: BMC Oral Health. 2019 Nov 1;19:234. doi: 10.1186/s12903-019-0931-1 (PMC6825342; doi:10.1186/s12903-019-0931-1)
Supplement: Supplementary file 1 — Additional file 1: Table S1. Multivariate odds ratios (ORs) and 95% confidence intervals (CIs) for tertiles of quality of life score (lowest tertile was used as the reference), according to predictors* and the interaction between age and number of teeth, among all participants (Weighted N = 1807). Results of regression analysis on tertiles of quality of life score. [file 12903_2019_931_MOESM1_ESM.docx]

**Additional file 1: Table 1.** **Multivariate odds ratios (ORs) and 95% confidence intervals (CIs) for tertiles of quality of life score (lowest tertile was used as the reference), according to predictors* and the interaction between age and number of teeth, among all participants (Weighted N=1,807)**

| **Predictors** | **Tertile 2 vs. Tertile 1 (ref.)** | | **Tertile 3 vs. Tertile 1 (ref.)** | |
| --- | --- | --- | --- | --- |
|  | **OR (95% CI)** | **p-value** | **OR (95% CI)** | **p-value** |
| *Age, years* | 0.99 (0.98; 1.00) | 0.212 | 0.99 (0.98; 1.00) | 0.141 |
| *Gender* |  |  |  |  |
| Male | 0.80 (0.65; 0.99) | 0.045 | 0.55 (0.43; 0.70) | <0.001 |
| Female (ref.) | 1.0 | - | 1.0 | - |
| *Level of education* |  |  |  |  |
| None/basic | 1.09 (0.78; 1.53) | 0.621 | 1.15 (0.78; 1.70) | 0.481 |
| Middle/technical | 1.10 (0.82; 1.48) | 0.540 | 1.03 (0.76; 1.39) | 0.869 |
| University (ref.) | 1.0 | - | 1.0 | - |
| *Smoking* |  |  |  |  |
| Current | 1.24 (0.84; 1.81) | 0.278 | 2.11 (1.55; 2.86) | <0.001 |
| Past | 1.00 (0.69; 1.48) | 0.989 | 1.02 (0.69; 1.49) | 0.977 |
| Never (ref.) | 1.0 | - | 1.0 | - |
| *Any diseases/conditions* |  |  |  |  |
| Yes | 0.93 (0.75; 1.16) | 0.544 | 1.40 (1.11; 1.76) | 0.005 |
| No (ref.) | 1.0 | - | 1.0 | - |
| *Number of missing teeth* | 1.14 (1.03; 1.27) | 0.014 | 1.18 (1.08; 1.29) | <0.001 |
| *Mean gingival index* | 1.54 (1.11; 2.11) | 0.009 | 2.27 (1.71; 3.01) | <0.001 |
| *Mean plaque index* | 0.97 (0.50; 1.87) | 0.920 | 1.57 (0.76; 3.25) | 0.227 |
| *Mean calculus index* | 1.09 (0.71; 1.68) | 0.690 | 0.88 (0.60; 1.31) | 0.533 |
| *Frequency of dental visits* |  |  |  |  |
| ≥ Once per year (ref.) | 1.0 | - | 1.0 | - |
| Never | 0.56 (0.29; 1.09) | 0.089 | 1.07 (0.49; 2.34) | 0.861 |
| Only when there is a problem | 1.09 (0.71; 1.68) | 0.684 | 1.58 (1.07; 2.32) | 0.021 |
| Missing | 0.72 (0.34; 1.50) | 0.373 | 1.47 (0.90; 2.40) | 0.125 |
| *Use of dental floss* |  |  |  |  |
| Yes | 1.03 (0.80; 1.33) | 0.811 | 0.95 (0.69; 1.30) | 0.724 |
| No (ref.) | 1.0 | - | 1.0 | - |
| *Location* |  |  |  |  |
| Kingston | 0.70 (0.51; 0.95) | 0.021 | 0.57 (0.34; 0.94) | 0.026 |
| Santo Domingo | 0.76 (0.54; 1.08) | 0.108 | 0.83 (0.57; 1.19) | 0.275 |
| San Juan (ref.) | 1.0 | - | 1.0 | - |
| *Age and Number of Missing Teeth Interaction* | 0.998 (0.996; 1.00) | 0.051 | 0.998 (0.996; 0.999) | 0.006 |

* Odds ratio estimates were obtained from a multinomial logistic regression model, with all listed variables as predictors and the three-level categorical OHIP score (tertile) as the outcome, using the lowest tertile (best summary quality of life score) as the reference.
